# The three *CYBA* variants (rs4673, rs1049254 and rs1049255) are benign: new evidence from a patient with CGD

**DOI:** 10.1186/s12881-017-0492-6

**Published:** 2017-11-13

**Authors:** Jinqiao Sun, Min Wen, Ying Wang, Danru Liu, Wenjing Ying, Xiaochuan Wang

**Affiliations:** Department of Clinical Immunology, Children’s Hospital of Fudan University, 399 Wanyuan Road, Shanghai, 201102 China

**Keywords:** Chronic granulomatous disease, *CYBB*, *CYBA*, Mutation

## Abstract

**Background:**

Chronic granulomatous disease (CGD) is an inherited immunodeficiency disease caused by the defect of NADPH oxidase. Mutations in *CYBB* or *CYBA* gene may result in membrane subunits, gp91phox or p22phox, expression failure respectively and NADPH oxidase deficiency. Previous study showed that three variants, c.214 T > C (rs4673), c.521 T > C (rs1049254) and c.^*^24G > A (rs1049255), in *CYBA* gene form a haplotype, which are associated with decreased reactive oxygen species generation. The study aims to confirm the three above mentioned variants are benign and report a novel mutation in CYBB gene.

**Methods:**

A patient with CGD and his family members were enrolled in the study. NADPH oxidase activity and gp91phox protein expression of neutrophils were analyzed by flow cytometry. Direct sequencing was used to detect *CYBB* and *CYBA* gene mutations.

**Results:**

The patient was diagnosed with CGD according to clinical and immune phenotype. The case has a novel homozygous mutation in *CYBB* gene and the above mentioned three variants in *CYBA* gene. The mutation in *CYBB* gene was confirmed to be pathogenic, and the three variants in *CYBA* gene to be benign.

**Conclusions:**

The study not only reported a novel mutation in *CYBB*, which results in CGD, but also confirmed the above mentioned three variants in *CYBA* are benign.

## Background

Chronic granulomatous disease (CGD) is an inherited immunodeficiency disease caused by the defect of NADPH oxidase [1]. There are seven NADPH oxidase isoforms, NADPH oxidase 1-5, DUOX1 and DUOX2, encoded by *NCF4*, *NCF1*, *NCF2*, *CYBB* and *CYBA* respectively [2, 3]. Mutations in *CYBB* gene, encoding the gp91^phox^ subunit, result in X-linked CGD that affects the majority of CGD patients (~70%) [4]. As expected from the genetics, the overwhelming majority of CGD patients are males. Protein p22^phox^ was discovered as the membrane subunit associated with NADPH oxidase 2, encoded by *CYBA* [5, 6] and more than 240 polymorphisms in the *CYBA* gene have been reported in dbSNP [7]. Some polymorphisms of the *CYBA* gene cause reactive oxygen species (ROS) generation failure and susceptibility to infection and autoinflammation, referred to autosomal CGD [8, 9].

Bedard et al. used Epstein-Barr virus transformed B lymphocytes from 50 healthy unrelated individuals to analyze their *CYBA* mRNA sequence and NADPH oxidase 2 dependent ROS generation [10]. Seven single-nucleotide polymorphisms (SNPs) were identified, yielding 11 distinct haplotypes which were grouped into seven haplogroups. In the study, a haplogroup containing all three major SNPs (c.214 T > C (rs4673), c.521 T > C (rs1049254), c.^*^24G > A (rs1049255)) in the *CYBA* gene showed a significant p22^phox^ deficiency and markedly reduced ROS generation compared to other haplogroups [10].

In our study, we found a CGD patient with a novel homozygous mutation in *CYBB* gene and the above mentioned three variants in *CYBA* gene. The other family members of the patient, who have no CGD, also have the same three variants in *CYBA* gene.

## Methods

### Patient

**Fig. 1:**
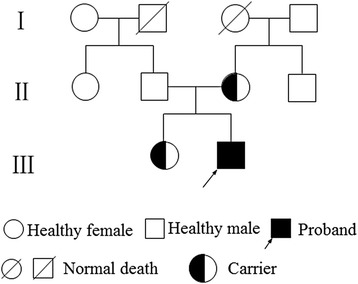
Family tree of the patient

The patient, a boy from a nonconsanguineous marriage, was referred to our hospital at 3 months of age. He had pneumonia and diarrhea when he was 20 days old, and then perianal abscess occurred. He received antibiotics and supportive therapy for 22 days, and discharged after condition improved. Shortly after discharge, he had fever again. His condition had no improvement after treatment in local hospital. Then, he was transferred to our hospital. Physical examination showed cervical and inguinal lymphadenopathy, hepatosplenomegaly, and perianal abscess. His father, mother, sister and other relatives all have no recurrent infection history. The family tree is showed in Fig. 1. All of the family members were detected.

### Neutrophil respiratory burst functional assays

The respiratory burst of neutrophils was assessed by hydrogen peroxide production using dihydrorhodamine oxidation (DHR) assay [11]. After stimulation with Phorbol-12-myrismte-14-acetate (PMA), neutrophils were immediately analyzed by using a FACSCalibur Flow Cytometer (Becton Dickinson, USA). The comparison was based on a stimulation index (SI), which was defined as mean channel fluorescence intensity of PMA-stimulated neutrophils over mean channel fluorescence intensity of unstimulated neutrophils [12].

### Detection of Cytochrome b558 protein

The presence of flavocytochrome b558 in neutrophils membrane was detected by Flow Cytometry using the monoclonal antibody (mAb) 7D5 (murine IgG1), provided by Toshio Miyawaki (Japan). In brief, neutrophils were stained with mAb 7D5 followed by fluorescent isothiocyanate-conjugated anti-mouse antibody. The stained neutrophils were run on a FACSCalibur Flow Cytometer (Becton Dickinson, USA) [11].

### *CYBB* and *CYBA* gene sequencing

Genomic DNA was isolated from peripheral blood mononuclear cells using the RelaxGene Blood DNA System (Tiangen Biotech, Beijing, China) according to the manufacturer’s instructions. Polymerase chain reaction (PCR) amplification of *CYBB, CYBA* was performed using synthetic oligonucleotide primers for each exon. After an initial denaturation for 5 min at 95 °C, 35 cycles of amplification were performed as follows: 95 °C for 30 s, 60 °C for 30 s, and 72 °C for 40 s. Final extension was performed at 72 °C for 7 min. PCR products were purified by Performa DTR Gel Filtration Cartridges and directly sequenced by ABI Prism BigDye terminators. Both strands were sequenced.

## Results

### Clinical characteristics

**Fig. 2:**
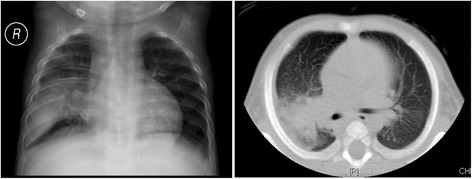
Chest radiograph and computed tomography scan of the patient at the age of 3 months, which revealed pneumonic infiltration and consolidation of the middle and lower lobes of the right lung

After hospitalization, the boy received antibiotics therapy. The chest CT scan showed pneumonia and pulmonary consolidation (Fig. 2). According to his clinical characteristics, we considered the boy might have CGD. So we did some laboratory test to check it. Finally, the diagnosis of CGD was confirmed by laboratory test. And then, we treated the boy with interferon-gamma, in addition to antibiotics. Diarrhea and pneumonia were controlled well. Until now, the boy is 2 years old and in good clinical condition.

### DHR analysis

**Fig. 3:**
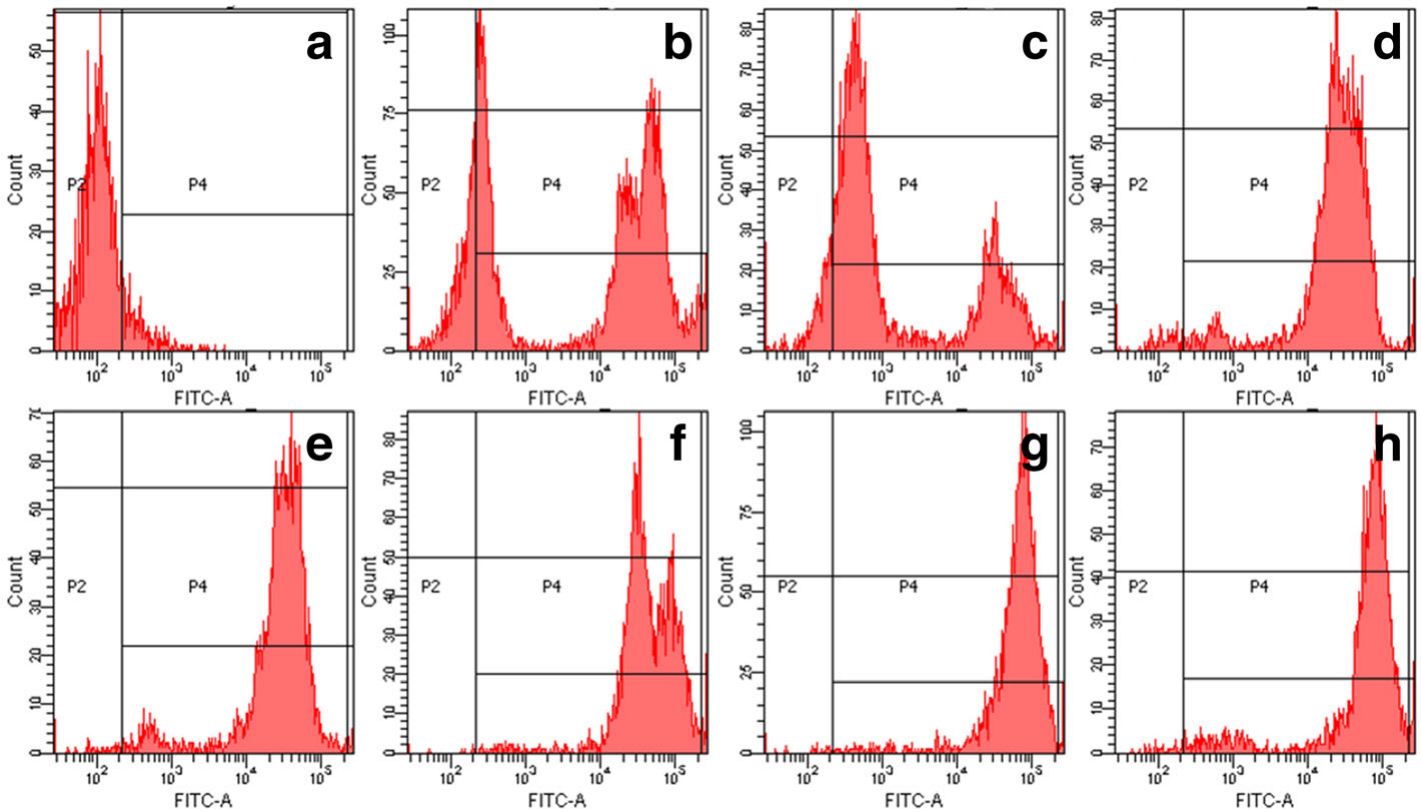
Respiratory burst of neutrophils. Hydrogen peroxide generation in neutrophils measured by dihydrorhodamine oxidation (DHR) analysis with Flow Cytometry. **a** the patient. **b** the patient’s sister. **c** the patient’s mother. **d** the patient’s father. **e** the patient’s paternal aunt. **f** the patient’s maternal uncle. **g** the patient’s paternal grandmother. **h** the patient’s maternal grandfather

The patient’s neutrophils were unable to produce superoxide after PMA stimulation. His mother’s and sister’s neutrophils showed a bimodal response pattern to PMA stimulation, superoxide production was higher than that in the patient, but lower than normal. His father and other relatives had normal neutrophil respiratory burst function (Fig. 3), consistent with healthy control. The results indicated that the patient has CGD and the mother and sister are carriers. To further confirm it, we did associated protein detection and gene analysis.

### Cytochrome b558 expressions

**Fig. 4:**
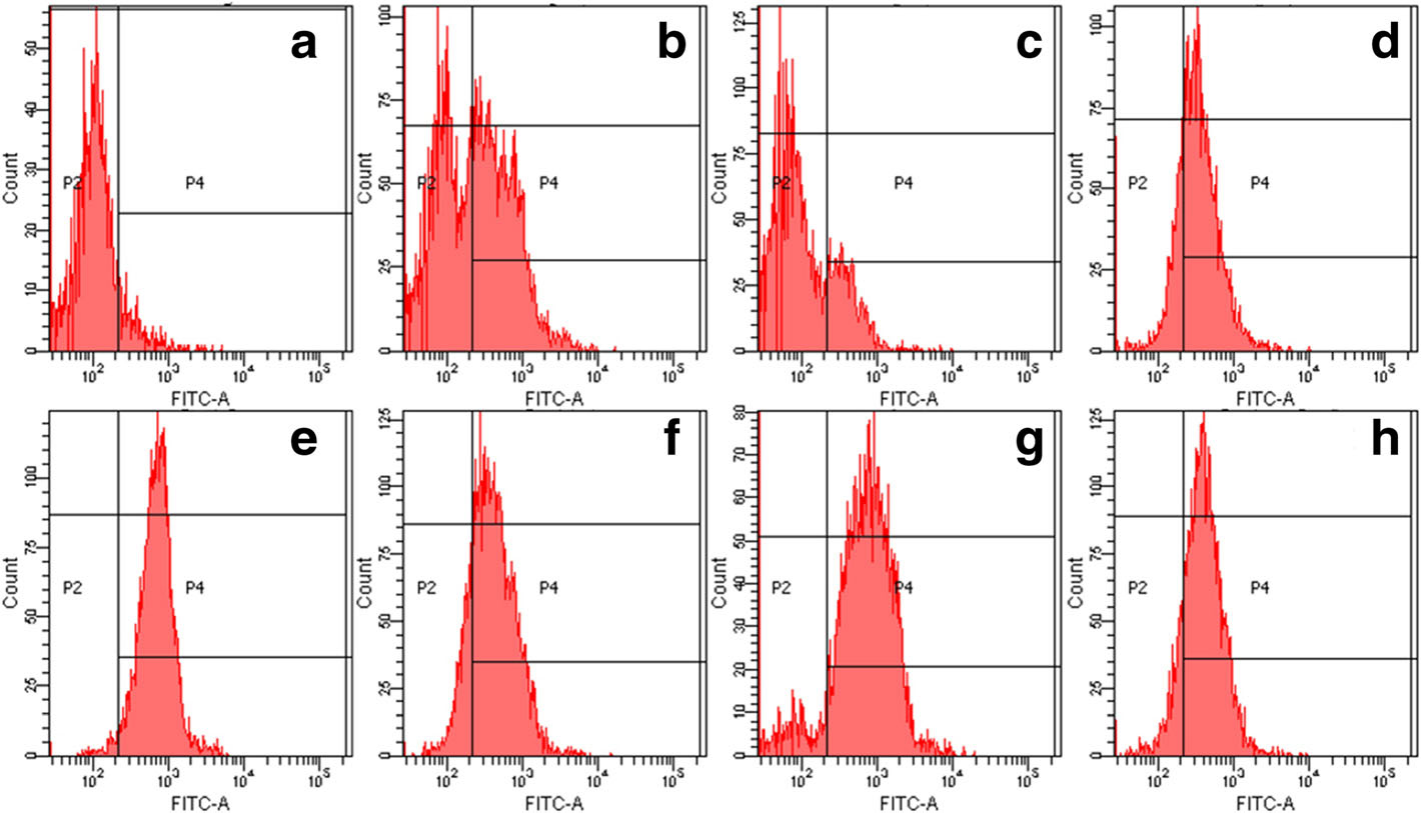
Cytochrome b558 expressions in neutrophils measured by Flow Cytometry. **a** the patient. **b** the patient’s sister. **c** the patient’s mother. **d** the patient’s father. **e** the patient’s paternal aunt. **f** the patient’s maternal uncle. **g** the patient’s paternal grandmother. **h** the patient’s maternal grandfather

Protein expressions closely resembled the DHR assay pattern. The patient^’^s flavocytochrome b558 was not expressed, suggesting a complete or near complete absence of gp91^phox^ or p22^phox^. His mother and sister showed a bimodal curve, indicating partial protein expression. His father and other relatives, who have normal SI, showed normal expression of flavocytochrome b558 (Fig. 4).

### Gene sequencing

**Fig. 5:**
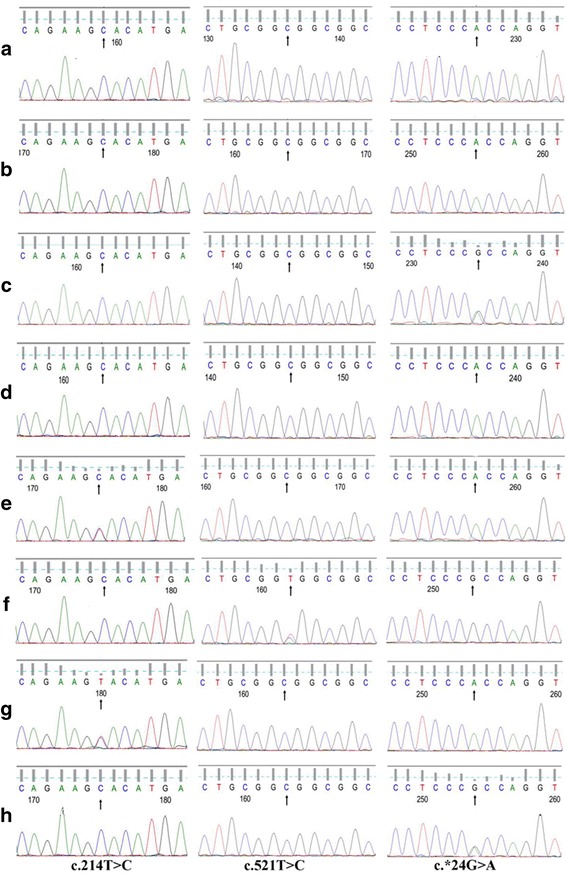
Gene sequencing of CYBA. Arrows indicate the mutation points. **a** the patient. **b** the patient’s sister. **c** the patient’s mother. **d** the patient’s father. **e** the patient’s paternal aunt. **f** the patient’s maternal uncle. **g** the patient’s paternal grandmother. **h** the patient’s maternal grandfather

**Fig. 6:**
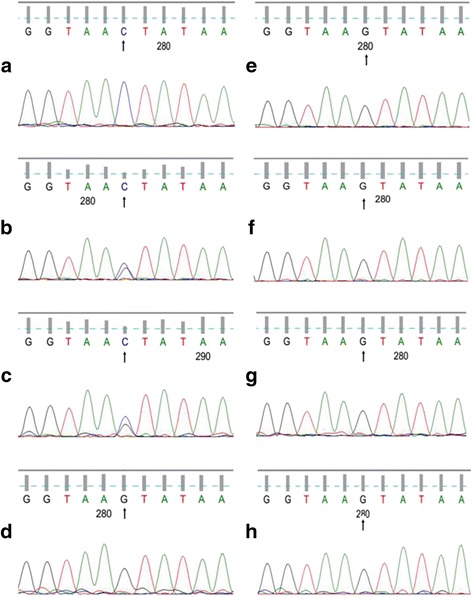
Gene sequencing of CYBB. The patient and his sister and mother have c. 141 + 5G > C mutation in *CYBB* gene. The other relatives are normal. Arrows indicate the mutation points. **a** the patient. **b** the patient’s sister. **c** the patient’s mother. **d** the patient’s father. **e** the patient’s paternal aunt. **f** the patient’s maternal uncle. **g** the patient’s paternal grandmother. **h** the patient’s maternal grandfather

Analysis of *CYBB* and *CYBA* genes was done. With respect to the reference sequence, the patient^’^s *CYBA* gene contains the above mentioned three variants, c.214 T > C, c.521 T > C, and c.^*^24G > A, and all the three variants are homozygous, as well as his father and sister. His mother has two homozygous SNPs, c.214 T > C and c.521 T > C, and one heterozygous mutation, c.^*^24G > A. Other relatives, including his maternal grandfather, his paternal aunt and grandmother all have two homozygous mutations and one heterozygous mutation. Besides, his maternal uncle has homozygous c.214 T > C mutation and heterozygous c.521 T > C mutation, no c.^*^24G > A mutation (Fig. 5). As to the *CYBB* gene, the patient has a novel homozygous mutation, c.141 + 5G > C, his mother and sister have heterozygous mutations at the same site and his father as well as all other relatives have normal *CYBB* gene (Fig. 6).

## Discussion

CGD is an uncommon inherited immunodeficiency occurring approximately between 1 in 200,000 and 1 in 250,000 live births [4]. CGD patients always suffer from recurrent bacterial and fungal infections including involvement of lung, lymph nodes, skin and gastrointestinal tract [13]. The X-linked forms are generally more prevalent in the world; about two-thirds of CGD patients show an X-linked inheritance, which is caused by mutations in the *CYBB* gene [14].

In this study, we suspected that the patient suffered CGD according to his clinical characteristics. The diagnosis of CGD was confirmed by defective neutrophil respiratory burst function and complete absence of flavocytochrome b558 expression. *CYBB* and *CYBA* gene sequencing was performed. We found a homozygous mutation, c.141 + 5G > C, in *CYBB* gene in the patient, and the same heterozygous mutation in his mother and sister. The *CYBB* gene of patient’s father and other relatives were normal. Two mutations at the same site were reported previously [15, 16]. They are c.141 + 5G > A and c.141 + 5G > T, respectively. They are not the same mutation as the mutation in our patient. The mutation we found is novel.

Interestingly, we found three variants (c.214 T > C (rs4673), c.521 T > C (rs1049254), c.^*^24G > A (rs1049255)) in *CYBA* gene in the patient. All the three variants are homozygous. His father and sister have exactly the same variants as the patient. Other relatives we detected all have these three SNPs, two sites are homozygous and the other one is heterozygous with the exception of his maternal uncle with one normal site. Because the patient’s father has the normal respiratory burst of neutrophils, the three variants should be benign. Previous study showed that there is a significant defect in ROS generation when these three SNPs form a haplotype [10]. Since lymphocytes are not the natural site for the respiratory burst, EBV transformed lymphocytes model with the three variants maybe have limitations and cannot reflect real state. Overall, the mutation in *CYBB* (c.141 + 5G > C) should be pathogenic from a combined analysis of DHR, protein expression and gene results.

## Conclusions

In summary, our study not only reported a novel mutation in *CYBB*, which cause CGD, but also confirmed the above mentioned three variants in *CYBA* are benign.

## Abbreviations

CGD: Chronic granulomatous disease
DHR: Dihydrorhodamine oxidation
ROS: Reactive oxygen species
SI: Stimulation index
SNPs: Single-nucleotide polymorphisms
